# Teaching seven principles for public health ethics: towards a curriculum for a short course on ethics in public health programmes

**DOI:** 10.1186/1472-6939-15-73

**Published:** 2014-10-07

**Authors:** Peter Schröder-Bäck, Peter Duncan, William Sherlaw, Caroline Brall, Katarzyna Czabanowska

**Affiliations:** Department of International Health, School for Public Health and Primary Care (caphri), Maastricht University, Postbus 616, Maastricht, MD 6200 The Netherlands; Faculty for Human and Health Sciences, University of Bremen, 28359 Bremen, Germany; Department of Education and Professional Studies, Centre for Public Policy Research, King’s College London, Waterloo Bridge Wing, Franklin-Wilkins Building, Waterloo Road, London, SE1 9NH UK; Department of Human and Social Sciences and Health Behaviour, Ecole des Hautes Études en Santé Publique, Avenue du Professeur Léon-Bernard, CS 74312, 35043 Rennes, France; Institute of Public Health, Faculty of Health Sciences, Jagiellonian University, Medical College, 31-126 Krakow, Poland

**Keywords:** Public health, Ethics, Education, Curriculum, Principle based ethics

## Abstract

**Background:**

Teaching ethics in public health programmes is not routine everywhere – at least not in most schools of public health in the European region. Yet empirical evidence shows that schools of public health are more and more interested in the integration of ethics in their curricula, since public health professionals often have to face difficult ethical decisions.

**Discussion:**

The authors have developed and practiced an approach to how ethics can be taught even in crowded curricula, requiring five to eight hours of teaching and learning contact time. In this way, if programme curricula do not allow more time for ethics, students of public health can at least be sensitised to ethics and ethical argumentation. This approach – focusing on the application of seven mid-level principles to cases (non-maleficence, beneficence, health maximisation, efficiency, respect for autonomy, justice, proportionality) – is presented in this paper. Easy to use ‘tools’ applying ethics to public health are presented.

**Summary:**

The crowded nature of the public health curriculum, and the nature of students participating in it, required us to devise and develop a short course, and to use techniques that were likely to provide a relatively efficient introduction to the processes, content and methods involved in the field of ethics.

## Background

### The context for teaching ethics in public health

Our purpose in this paper is to explain and discuss a framework for a university-based short course in public health ethics. The framework has been developed and employed now in several European universities and schools of public health, including the Ecole des hautes études en santé publique (France), Maastricht University (the Netherlands), and Bielefeld University (Germany). We begin by discussing some aspects of the context for teaching public health ethics that were important in our deliberations on why and how to engage in such teaching: and which formed fundamental starting points in the development of our framework. We then move on to explain and elucidate the mid-level ethical principles that form the content cornerstone of the framework; and the educational approach that it attempts to model. Crucially, we are not setting out simply to offer a description of what we have done but instead to analyse, discuss and ultimately attempt to justify both content and educational approach.

Public health professionals are frequently called upon in their daily practice to make both explicit and implicit choices that extend beyond the objective and practical and into the contested and ethical [1]. Balancing and coming to conclusions about the rights and duties of individuals, communities, populations and governments with regard to protecting and maintaining health is in many ways the central, deeply complex task of public health work [2]. Yet at the same time, evidence strongly suggests that public health professionals often receive little training and guidance on how to reach decisions informed by careful ethical thinking and become confident in a moral sense about the ‘trade-offs’ they are frequently required to make in practice [1, 3].

Often facing difficult decisions without adequate training and preparation in an ethical sense is, therefore, a feature of the public health context that motivated us to think carefully about addressing this subject through teaching and learning. We were well aware of the pressures contributing to this state of affairs. These included the already crowded nature of the public health university curriculum and the difficulty of simply employing dominant conceptions of ‘medical ethics’ to a field with (at least in many respects) quite different concerns and priorities [4]. Moreover, knowledge about, and evaluation of, ethics education in teaching and learning about public health (in contrast to ethics in medical education) remains comparatively scarce [3, 5].

Given these contextual issues, we were faced with an important set of questions: on what sorts of foundations should teaching and learning about public health ethics be based? How should teaching and learning in this field be enacted? What are the justifications for particular educational approaches? How can hard-pressed practitioners be sensitised to the idea that ethics permeates everything they do and that ultimately their enterprise is a moral one?

## Discussion

### Content foundations for teaching and learning in public health ethics: the choice of mid-level principles

Teaching and learning in public health ethics involves making choices about *what* to teach, as well as *how* to teach it. The brief description and discussion of context that we have so far engaged in leads us towards beginning to describe and discuss our choices in relation to what we actually teach about.

The starting point for our discussion about the content of public health teaching is our belief that those engaged in it (both as teachers and learners) need to discriminate between and evaluate a complex range of normative judgments. Those who are working in public health are rarely doing so without having taken up normative positions on the purpose of the enterprise and the nature of its particular interventions and activities. They are operating with certain beliefs about, for example, the kind of society that public health should be aiming to reproduce [6] or about the sorts of ways in which individuals, communities or populations should lead their lives [7]. So an important outcome of teaching and learning in public health ethics is the capacity to make reasoned evaluations of the range of normative beliefs and values at work in the field.

As a discipline, ethics is also itself (at least in part) normative. It is about identifying and attempting to agree the importance of particular values (or kinds of values) [8]: and how and why separate values might influence decisions and choices about action. So those involved in teaching public health ethics have a further task of evaluation and discrimination: between the competing normative systems and judgments of moral philosophers themselves.

In fact, the tasks of evaluating normative beliefs within public health on the one hand, and normative judgments made by philosophers on the other, are complementary, indeed intertwined. The foundations of value-based decisions in public health (as with the broader field of health care and medicine more generally) lie in moral philosophical conceptions of what is valuable [9]. This leads us to the view that our framework for public health ethics teaching and learning should be based on a set of mid-level ethical principles, and critical appraisal and evaluation of these principles.

What do we mean by mid-level principles and why have we chosen them to form the central content of our framework? Such principles represent normative thinking that might stem from more than one moral philosophical theory and thus can be connected back to several theories. They are at the mid-point of a hierarchy that at its top is formed of overarching theories that attempt to explain and justify particular normative positions (for example, deontology and the pre-eminence of duty in moral consideration, or theories that focus on the importance of consequences in ethical deliberation); and at its bottom comprises a range of particular rules (expressed, say, through devices such as codes of conduct).

We argue here that because the principles are mid-level, and hold connections both with a number of normative theories and with the multiple prescriptions of codes and guidelines, they therefore garner wide acceptance [9]. The importance of individual principles such as we are advocating is also demonstrated by their being reflected in significant parts of the bioethics and public health ethics literature [9–13]. Thus the selection of these principles finds support and reflects positive experience in practice, as one of the authors of this current paper has established in previous work [14]. Equally, such principles may not command complete acceptance and can be challenged [15], making them highly useful in terms of encouragement for reflection and debate.

This combination of acceptability on the one hand and the potential for helpful challenge on the other provides justification for our choice of such principles. Given the major task of ‘squeezing ethics in’ to the crowded public health curriculum, employing them to provide the content foundation of our course framework allowed the opportunity for fairly swift appreciation of their relevance in the short time that we had available; while also proposing them as stimulators of more lengthy reflection, possibly undertaken outside and beyond formal class hours. Our choice aligns with the deliberations of the seminal Belmont Report:

‘[R]ules often are inadequate to cover complex situations; at times they come into conflict, and they are frequently difficult to interpret or apply. Broader ethical principles will provide a basis on which specific rules may be formulated, criticised and interpreted’ [16].

This justification of the use of mid-level principles as the content foundation of our teaching, and their location in a hierarchy of normative ethical theorising and judgment, leads us briefly to describing and discussing the principles themselves. Because of their place in the hierarchy, we need to note that an important anticipated outcome of our teaching and learning will be that students should be able to link the principles to overarching theories that exist ‘above’ them. Our particular concern is to encourage students towards recognising, understanding and critically appraising the principles’ connections to consequentialist theories (the value of an action lies in the good or bad consequences that it produces) such as utilitarianism; and deontology (the worth of an action lies prior to any consideration of its particular consequences and instead on its performance as a *duty*).

We follow Beauchamp and Childress [9] not only in their account of mid-level principles but also as conceiving of them as being *prima facie*: each of equal weight at the outset of moral deliberation. Naturally, during the course of such deliberation, it is both possible and likely that a particular principle or principles will assume more or less importance. Thus the *prima facie* status of the principles, in our view, supports the process of careful ethical deliberation and reflection; answers are not ready made from the outset and choices have to be formulated.

There are seven principles that form the content grounds of our teaching framework:

#### Non-maleficence

The principle of non-maleficence – do no harm – asserts that a health care professional should act in such a way that he or she does no harm, even if her or his patient or client requests this [9]. This principle is the first to be proposed because of its historical antecedence; it is related to the famous Hippocratic ‘*primum nil nocere*’– first of all, do no harm’ of medical ethics, although not identical to it [9, 17, 18]. Within public health policy and practice, there are often occasions where degrees of harm are ‘traded off’ against the possibilities of greater harms, or perhaps positive benefit: for example, banning smoking in public places may cause harm to individual smokers but will prevent greater harms (and arguably produce benefit) through acting as a general disincentive to smoking among the wider population. Consideration of the non-maleficence principle shifts – at least – the burden of proof to those exercising potentially harmful behaviour that they are justified in doing so.

#### Beneficence

The obligation to produce benefit, for individual patients or clients, as we have implied above, is intimately connected to non- maleficence. Its apparently self- evident importance marks it out as the other core principle within the Hippocratic tradition: physicians should heal and help their patients, according to the physician’s abilities and judgment [19]. The distinctive difference between the principle of non-maleficence on the one hand and that of beneficence on the other lies in the fact that the former frequently – but not always – involves the omission of harmful action and the latter active contribution towards the welfare of others [9].

#### Health maximisation

Non-maleficence and beneficence can be understood in both deontological and consequentialist terms. Yet as principles they do not seem to go to the core of public health values. This is at least partly because of their tendency to be associated with, and used in trying to analyse, individual professional-client encounters. Even when following beneficence and non-maleficence in these individual encounters, it does not necessarily mean that population health is maximised, as the population is not at all within the focus of these micro- encounters. In the field of public health, the primary end sought is the health of the broader constituency of the public and improvements to this are the key outcome used to measure success [10]. In fact, the maximisation of population health, on the one hand, and beneficence and non-maleficence, on the other hand, can come into conflict.

One way of conceiving of the moral impulse of beneficence in public health terms is therefore to understand the ethical imperative to produce benefit in a wider sense and to talk of the obligation to ‘social beneficence’. Here we are thinking of the idea that public health professionals have an obligation to maximise health in the populations for which they are responsible. In fact, our preference is for the ethical principle underscoring this obligation to be referred to as one of health maximisation. This is because we need to be more specific than simply saying public health professionals have a duty to produce benefit (implied by the idea of ‘social beneficence’). What constitutes benefit (at both individual but especially at population level) is subject to dispute and may not necessarily be understood as ‘health’. It seems perverse to claim that public health professionals are primarily interested in other kinds of benefit over and above maximising health and opportunities for health; thus a specific principle of health maximisation, we argue, needs to constitute the third of the mid-level principles that form the content grounds of our short course teaching and learning. Of course, none of this is to deny the disputability of the concept of health, and the possibility of profound disagreement about what exactly it is that we are attempting to maximise [20]. There is a strong requirement to focus on maximising (population) *health* rather than on wider concepts of the “(common) good” (whatever is understood by this), which might well be outside the scope of public health. We will return to this point later in our discussion.

#### Efficiency

There will always be more health need than resources to deal with that need. Literally all public health systems (and health care systems) worldwide lack resources. These two statements prompt the advocacy of a moral duty to use scarce health resources efficiently. This duty exists at least partly because efficient use will enable public health professionals to produce more health benefit for greater numbers of people. So a moral principle of efficiency would demand, for example, the use of the evidence base and the performance of cost-benefit analyses to decide what should be done and how to do it.

As with the problematic of agreeing on the exact nature of the ‘health’ that we are supposed to be maximising in the previous principle, however, there is an equal difficulty here. ‘Efficiency’, along with associated notions such as ‘cost’ and ‘benefit’ are complex matters. For example, in considering the cost and benefit of undertaking (or not undertaking) a particular public health intervention, are we limiting our views of these things simply to the health sector or to the effect of the intervention on the wider social fabric and governance of public services? Moreover, it is conceivable to imagine limited or no action in the public health field as constituting ‘efficiency’ in the sense of negligible resource input yielding negligible returns but the cost-benefit ratio appearing reasonable in solely economic terms. Here we need to emphasise that the principle of efficiency has *moral* applicability, which needs to be disentangled from other considerations of efficiency, such as economics. (Efficiency is frequently linked to notions of ‘effectiveness’. We chose not to include ‘effectiveness’ as an explicit principle because it is somewhat implicit in the principle of health maximisation, and the strong sense this particular principle conveys that ethical public health action should naturally entail improvement in population health).

#### Respect for autonomy

The paternalistic benevolence contained in the principles of non-maleficence and beneficence is strongly tempered by the emphasis on respect for the autonomy of the patient who the health care professional is seeking to serve [9, 21]. The principle of respect for autonomy extends, however, beyond the confines of individual health care; it is crucially important within the public health context. The frequent focus of public health on benefit for populations holds the potential for concern with individual welfare to be side- lined. Embedding respect for autonomy firmly within public health ethics teaching and learning provides a fundamental reminder that every person has a high value – *qua* her or his autonomy – and cannot merely be treated as a means to the end of others’ good. Despite this, however, the tension between individual rights and broader conceptions of public benefit is a profound one for public health as a field of practice. This tension, and the relative command that such broader conceptions of benefit often seem to possess, leads us to assert that in cases where autonomy restriction for wider public health goals is being contemplated (e.g. legislation banning smoking in public places or limiting movement during periods of contagion), the burden of proof for doing so needs always to lie with those advocating restriction.

#### Justice

It is equally possible to conceive of the principle of justice (sometimes ‘social justice’) as having grounds in the fundamental value of human autonomy. Because as humans we all have (or should have) autonomy, we all have (or should have) equal moral worth. Thus, proposals for the unequal treatment of people again require the burden of proof. Justice, to the contrary, demands equal opportunities. This also includes a fair distribution of health outcomes in societies, which is often discussed in terms of public health as ‘health equity’. In a very prominent conception of justice in the context of health, Daniels [13] considers health equity thus a matter of fairness and justice. Under Daniels’ conception of justice, health inequalities are unfair and unjust – and thus in conflict with health equity – if the socially controllable factors that lead to health are not distributed in such a way that the health of all citizens is protected or restored as much as possible.

Given the essential importance of health in the formation and development of every aspect of our equally valuable human lives – what Boorse [22] describes as ‘species typical functioning’ – we owe each other equal access to health goods and positive determinants of health [13]. Justice is also the principle that covers normative aspects that are often discussed in the terminology of solidarity and reciprocity. Justice does so by giving an answer to the question of what we owe to each other [13]. To have a concise set of principles, we focus only on justice.

#### Proportionality

Our seventh and final principle differs somewhat from those preceding it. As a principle, proportionality is certainly normative. It demands that in weighing and balancing individual freedom against wider social goods, considerations will be made in a proportionate way. According to Childress *et al.*, proportionality:

‘Is essential to show that the probable public health benefits outweigh the infringed general moral considerations […]. For instance, the policy may breach autonomy or privacy and have undesirable consequences. All positive features and benefits *must be balanced* against the negative features and effects […]’ [10: 173, our italics].

However, proportionality is also a *methodological* principle. In a manner different to the principles we have so far discussed, it forms the basis for casuistic reasoning in relation to problems of individual welfare *versus* collective benefit in public health. Singer *et al.* [11], for example, argue that revelations of Chinese ethnicity in the Canadian outbreak of SARS demonstrate the need for fundamentally careful consideration before the release of private information in cases of pandemic disease. Beyond this, the balancing of private goods and public interests provides a way into debating many of the central problems of ethics in public health policy and practice such as resource allocation, the location of individual responsibility and foundational rights in the sphere of health and health care. It is this idea of debating the proportionality of interventions, and the help it offers in advancing understanding of situations, that leads us to our conception of the principle as partly methodological. Even though a methodological principle, it is normative nevertheless, and thus we include it in our concise set of principles: as with the other principles so far discussed, it contains essential *prima facie* moral guidance for public health practitioners.

### Process foundations for teaching and learning in public health ethics: case studies and problem based-learning

Having outlined and discussed the seven principles that form the content basis of teaching on our short course, we turn now to describing and discussing the processes for teaching and learning related to these content foundations. Our approach can be summarised as the use of case studies to stimulate debate and discussion around the principles that we have identified and discussed. The intention of case study-based debate is to allow reflection and awareness that ethical difficulties in public health are not ‘black and white’; we cannot expect easy answers, or possibly any definite answers at all [9, 23, 24].

#### Why case studies?

Case studies in this context are short narratives describing a real-world or at least realistic example of a professional ethical dilemma. Case studies have a central role in the process of teaching and learning that aims to build the capacity of moral awareness and discrimination. The use of case studies has been widespread and successful in various areas of medical ethical education generally [25] and bioethics more particularly [26]. They also have a history of success in public health, in particular public health ethical-scientific discourse [27].

The narratives embodied in case studies help to identify and illustrate ethical difficulties. Case studies, with their obvious focus on practice and practical examples, can help to unpack difficulty that is simply impossible through purely abstract ethical reasoning or generalised philosophical examples. They also offer the possibility of genuinely inter-disciplinary dialogue between public health practitioners and moral philosophers (both likely to be involved in ethics-related teaching and learning in this field), at least partly because they are ‘acceptable currency’ to both sets of people. The requirement for inter- disciplinary dialogue extends, moreover, beyond simply public health practitioners and moral philosophers to a range of others (for example, politicians and policy makers) simply by virtue of what public health is and what it tries to do.

Case studies are not simply ‘administered’. Their form demands, and their function yields, dynamic group discussions in which the participants’ specific professional and personal experience can be brought to bear on the problem highlighted within the case concerned.

An important benefit of a case study-type approach centrally embedded in public health ethics teaching and learning is that it allows access to an enormous range of sources and experience. There is perhaps a tendency to think of case studies as artefacts solely designed by those charged with the teaching and learning process. Of course, the development and use of case studies designed by those teaching short courses in ethics is important. But student-generated experience as material for case studies is equally, if not more, valuable because it is rooted in the professional lives of learners. Sources such as books (both fiction and non-fiction) and films are also rich veins that can be tapped in the search for source material for ethics-related case studies [28, 29].

#### Case studies as an aid to problem-based learning: the schedule for a short course in public health ethics

Having described the value of case studies for public health ethics teaching and learning in terms of their relevance, applicability and capacity to encourage inter-disciplinary dialogue, we now turn to exemplifying a schedule for a short course in this area. In doing so, we start to draw out the central importance of problem-based teaching and learning in our schema. (Please see Table 1 for a summary of this schedule).

**Table 1:** **Phases of a public health ethics course**

Phase	What	How	Who	Time
1	What implications can different understandings of “health” and “public” have for public health ethical discourses? What is ethics and how can it be useful for public health practice?	(Interactive) Lecture	Facilitator-led	3-4 hours (opportunity to go into greater depth with normative scope and ethical foundation of principles)
	Introduction of: Ethical principles, checklist, scheme for ethical judgement formation (Table 2).			
2	Exploring and critically examining possible scenarios for resolving a case together	Group discussion, led by facilitator	Facilitator and all students	
3	Solving a case study	1) Identification of the ethical challenge and conflict, 2) phrasing it in ethical language, 3) suggesting a solution by developing an ethical judgement based on an ethical argument (cf. Table 2).	Groups of students (4-6 in one group), facilitator goes from group to group to check if there are questions.	At least 1-2 hours
4	Presentation of results	Presentation in class by representative(s) of groups, discussion of group results.	Students; facilitator participates in discussions	1-2 hours (with more lengthy discussions)

In a first phase, our course begins with an introductory discussion focusing particularly on the concept of public health. What do we mean by this and in particular, what do we mean by its two constituent words, ‘public’ and ‘health’? Understanding these terms has essential relevance to ethics-related discussion of the field. The term ‘public’, for example, could be understood as the subject of action (the public being represented by public institutions) or as the object of action (someone acting to protect or improve the public’s health or pursuing a public good) [14, 30, 31]. Furthermore, different conceptions and criteria of health exist. Equally, students need to be encouraged to develop an awareness that how they understand ‘health’ (both generally and in the context of public health particularly) will have implications for how they frame ethical discourses and move towards resolving moral problems. Yet the task of defining and describing ‘health’ itself is complex and ridden with competing values [32, 33]. As a consequence, the concept of public health can also be interpreted differently [34, 35]. Encouragement is made towards the idea that our understanding of the term ‘public health’ and its constituents ’public’ and ‘health’ is likely to be neither wholly objective nor completely neutral; our talk is always (at least partly) driven by ideology [13, 20, 22].

Debate about the nature of health and its relation to allied concepts such as well-being, illness, disease and disability is important both to help frame and understand the discussions that follow; and also to prompt at the earliest stage of the course dialogue between its participants. Our experience is that those undertaking the kind of course we describe may enter it believing that they broadly share similar conceptions of ‘health’ and ‘public health’. This may of course be true, but we have found that going back to first principles in the way that we have described is often a means to exposing differences in understanding, which warrant fruitful exploration as part of the ethics-focused debate that follows.

After this introductory session, we move on to begin discussion of ethics, focusing on its capacity to inform decision-making [36]. Our concern is to present ethics as a systematic field of study and a major historical contributor to the development and shaping of society. We also attempt to explain and clarify the *normative* character of much ethical thinking, a central feature of its character that is likely to differ from other fields and disciplines with which participants may be more familiar. Although of course we have so far argued that much public health practice is predicated on normative assumptions and beliefs, this is not often rendered visible. Perhaps the greatest difference between the discipline of ethics and other potential disciplinary contributors to the public health curriculum lies in the normative focus of ethics being *explicit*.

Ethical argument and resulting positions are generally driven by the belief that this is the way that things *ought to be* in the world [37]. This is the essential meaning of normativity in ethics. Public health practitioners – our course participants – may struggle with the move from ‘is’ to ‘ought’ that is this central characteristic of normative ethics. They are likely to be much more familiar with fields and disciplines in which evidence is developed and presented: and arguments may be made for a particular position; but normative declarations are not (or at least not often) made in relation to these processes. To take a brief example, a public health practitioner may, in his or her practice or other study, have gathered evidence for the existence of inequalities in health. They will most likely have views on what this evidence implies for the lives of individuals and populations. But it is unlikely (other than perhaps in a personal sense), that they would have been required to develop a normative argument related to inequalities (e.g. health inequalities should be regarded as morally unacceptable when the determinants of these inequalities are avoidable). Ethics easily assumes this latter kind of position, but reaching it may be unfamiliar for our participants.

This example emphasises the importance of our problem-based learning approach within this course. By confronting our participants with a problem and asking what *should* be done, and, importantly, what we need to explore and understand better to be able to justify such action, they are guided, or hopefully guide themselves, through an essential process. This is the process that requires them to account for, and come to conclusions about, not simply their knowledge and understanding of the issue being considered, but also their experience (or potential experience) of that issue. This connection of knowledge, understanding and experience is likely to yield different positions and conclusions than one founded simply on cognition. It is likely to allow and facilitate the adoption of normative positions (this is what I should do, or what I should believe), which can then be subject to scrutiny. This is because we are ‘allowing’ the expression of values through emphasising the importance of experience (that on which, in large part, our values are founded) [38].

At the same time, students will need points of reference and justification for the ethical positions that they are constructing through their consideration of problems and cases. Thus, the step of our teaching and learning is to introduce the principles (as described and discussed above) that provide normative guidance (and which of course have been developed through the lengthy application of careful thinking related to the nature and purpose of health care, among other areas of human endeavour). Developing thinking about practical experience (either one’s own, or in a vicarious sense) and striving for justifications or actions – or omissions – forms the essence of ethical deliberation. This is practiced in discussing a case together (see phase 2, Table 1).

As we made clear in the introduction to this paper, balancing possible courses of action and coming to conclusions about what should be done is a key feature of professional life in public health. These conclusions are not simply (or even most importantly) practical ones; they are ethical. Our interest in developing the course we are describing and discussing emerged from a belief that frequently there is little or no training or preparation for ethical thinking and understanding in the process of the formation of public health professionals. In our course up to this point, we have demonstrated the need for this understanding, proposed both tools (the principles) and methods (the use of case studies and the application of problem-based learning in the context of the methodology of ethics) and are now at the final stage of applying these.

In this next phase, students are divided into small groups of between four and six, depending on overall group size. Each group receives a different case study, which illustrates an underlying ethical problem or conflict. (Please see Case study: Maria Morales for one example case study used by the authors in their teaching and learning). They also receive a hand out that is their ‘toolbox’ for approaching and dealing with the problem. This contains a summary of the principles that we have elucidated and a checklist/aide memoire for their application. (Please see “Principles checklist/aide memoire”).

### Case study: Maria Morales

Maria Morales, head of the “Infectious Disease Control” unit of the Ministry of Health of the State X, is asked by her minister to make a suggestion if measles immunisation should be made mandatory in their region as recently 2 children died after a measles outbreak. State X has an insufficient immunisation rate (1^st^ dose 70%, 2^nd^ 55%). Maria finds out that *obligatory* measles immunisation is effectively implemented in regions in Hungary and the Czech Republic. She knows her minister is taking her advice most seriously. What should she do? [39].

### Principles checklist/aide memoire

- *Non-Maleficence*
  - ✓ Will no one be harmed by the proposed intervention?
  - ✓ Are especially children prevented from harm?

- *Beneficence*
  - ✓ Is the intervention of any good to every single person taking part in this intervention?
  - ✓ Overall, for both non-maleficence and beneficence, is it possible to assess whether more benefit than harm is produced by intervening (or not intervening) and, if so, on what side (benefit or harm) does the equation finally fall?

- *Health Maximisation*
  - ✓ Is the proposed intervention effective and evidence-based? Does it improve population health?
  - ✓ Does it have a sustainable, long-term effect on the public’s health?
  - ✓ Is there a community added value to the proposed intervention?

- *Efficiency*
  - ✓ Is the proposed intervention cost-effective?
  - ✓ Awareness of scarcity of public money; saved money can be used for other goods and services.

- *Respect for Autonomy*
  - ✓ Does the intervention refrain from employing coercion and manipulation? Does it foster free choice?
  - ✓ Is there really ‘informed consent’ to take part in the intervention?
  - ✓ Is self-responsibility not only demanded but also possible for every person?
  - ✓ Are privacy and personal data respected?
  - ✓ If the intervention is paternalistic, is this justifiable?
  - ✓ Does the intervention promote the exercise of autonomy?

- *Justice*
  - ✓ Is no one (including third parties) stigmatised, discriminated against or excluded as a consequence of the proposed intervention?
  - ✓ Is the institution proposing the intervention publicly justified and acting transparently?
  - ✓ Is the proposed intervention not putting sub-populations at risks of being excluded from social benefits and/or universal access to health care?
  - ✓ Does the intervention exacerbate social and health inequalities (inequities)? Does it fight inequalities (inequities)?
  - ✓ Does the intervention consider and support vulnerable sub-populations (e.g. migrants)?
  - ✓ Does the intervention promote rather than endanger fair (and real) equality of opportunity and participation in social action?
  - ✓ Does the intervention refrain from eroding a sense of social cohesion and solidarity?

- *Proportionality*
  - ✓ Is the intervention the least infringing of possible alternatives?
  - ✓ Are costs and utility proportional?

Each small group discusses the case that it has been given. They can follow the detailed steps as presented in Table 2. Participants are asked to:

**Table 2:** **Steps of applied ethical reasoning; own source, inspired by** [40–42]

Steps	Selected questions and issues raised by the example case study “Maria Morales”
1. *Identify and frame in own words*: What is the underlying moral conflict?	Can a parents’ right to not have an intervention done with their child be overridden by the state (for someone else’s good)? Furthermore: Can parents exercise their will on behalf of their children?
2. *Identify and frame in ethical words*: Which ethical principles are relevant, how can they be specified and might they be in conflict to each other?	Overall, the principles respect for autonomy and health maximisation seem to be affected and seem to mutually exclude each other. But one also has to ask whose autonomy is at stake. Parents’ autonomy – but what about the future autonomy of children? Furthermore, the immunising doctor might be indecisive whether to advocate for autonomy, health maximisation or non-maleficence.
3. *Zoom further in*: Do I have all relevant information? Can I get more background information to understand all particularities?	What are the potential side effects of measles immunisation? How severe are measles for children? About how many persons (to be vaccinated against their parents’ will) can be protected, which effect would such an immunisation programme have on the incidence of measles and which side-effects could actually be prevented?
4. Are *alternative solutions* feasible with less moral issues/costs?	Can there be alternative approaches to mandatory measles immunisation? Can one raise immunisation rates by informing, advertising, setting incentives for parents?
5. *Further Specification*: Do the specifications change with more information?	If there are alternative ways that are less infringing on the respect for autonomy but rather support the health maximisation and the protection of those who cannot be immunised (ensuring non-maleficence), then these alternatives have a higher moral value.
6. *Weighing*: Are all conflicting principles and their specifications still of equal value?	If other measures (incentive setting, education campaigns for immunisation) can be successfully implemented elsewhere, mandatory immunisation seems less necessary. Yet, autonomy of the parents (who are safeguarding the autonomy of their children) attains even more weight.
7. What do I *conclude* from the specification and weighing? What would be my *solution* to the problem?	Mandatory measles immunisation would – in this very particular situation – not be necessary in order to achieve best health and given that it would infringe autonomy of parents (and allegedly of children), it should not be applied.
8. *Integrity*: Can I personally accept the conclusion drawn?	It seems to be a suitable solution to – at least first – try other measures, rather than being in charge of forcing parents and children to have children’s bodies ‘invaded’ against their ‘guards’ will.
9. *Act and convince:* I act according to my judgment and convince colleagues and others also based on ethical reasoning.	I try to find resources within my professional budget and start action to promote immunisation with other means.

- Identify as specifically as possible what they believe to be the ethical challenges and potential conflicts within the case;
- Frame these challenges in explicitly ethical language (i.e. according to the principles and other normative moral theory so far discussed within the course and contained in their ‘toolbox’);
- Suggest a ‘solution’ or otherwise a way of dealing with the case through the development of an ethical argument that again uses the resources of the ‘toolbox’.

At this last stage of the small group work phase, the groups formulate a justification for action that both elucidates the normative processes that have led them to their conclusion; and present an argument as to how and why they have rejected and would deal with alternative possible normative positions. (For example, in the presented case study “Maria Morales”, population benefit *versus* parental autonomy). Each small group in turn presents their justification and anticipation of counter-argument to the group as a whole in a plenary session. The emphasis in this session is not simply on exposition but also on continuing and developing the ‘dialogue of argument and understanding’ that the small group work has begun to generate.

## Summary

### Developing the ethical persona of the public health practitioner

In this paper, we have presented and justified our short course framework for ethics teaching and learning in public health. Our premise was that public health practitioners are frequently faced with difficult situations in which they have to make decisions with explicitly moral dimensions and yet they receive little training in the area of ethics. The crowded nature of the public health curriculum, and the nature of students participating in it, required us to devise and develop a short course, and to use techniques, that were likely to provide both a relatively efficient introduction to the processes, content and methods involved in the field of ethics; and make use of the understanding and experiences of our likely participants.

Our aims in presenting the framework have been modest. Primarily, they are to raise awareness of ethical issues within the practice of public health; and to provide a ‘toolbox’ to support thinking and reasoning (and possibly decision making) on the part of public health professionals in training. The modesty of these aims stems, as we have made clear, from a keen pragmatism about what can actually be achieved in this context.

Of course, this is not to exclude the fundamentally important proposition that this kind of introduction can only ever provide a ‘snapshot’ for participants of an enormous and (we believe) essentially interesting territory. In view of the fact that the endeavour of teaching ethics is currently a work under development in most European schools of public health, the approach described and discussed here can perhaps be used as a ‘minimum standard’ curriculum for teaching and learning in this area. We argue, however, that the limitation of our highly specific approach to a deeply complex area is outweighed by its forming at least the basis for independent thought, which we hope will extend well beyond the time boundaries of the short course itself.

We would hope that our short course model, or something approaching it, could be used until it is possible for programme directors to be able to designate more space for ethics modules in their programmes and until more fitting curricula, broadly encompassing ethics, are made available. Our approach in this short course framework has been to develop the realisation that independent ethical thought is possible, but that circumstances require guidance and direction. In this respect, we suggest that although the course may be considered as being partly akin to what Dawson [43] has described as ‘outside in’ ethics – the idea that principles act from the outside and guide practitioners in their ethical behaviour – it also sows the seed through case study deliberation for the emergence of ‘inside out’ (also from Dawson) – oriented ethical practitioners.

‘Outside in’- oriented ethics has value, but also has limitations, especially in the regard that, without principles being there, or being readily applicable (which may frequently be the case given the complexity of public health practice), the practitioner is rendered more or less helpless. While our course is based, as we have described, on principles, we have tried to make clear that it is not in any sense about ‘outside in’ rote learning of these principles. Our case study and problem-based learning approach allows the possibility of ‘inside out’ ethics. We encourage through our methods the development of independent ethical thinking on the part of those involved in public health. The essence of an ‘inside out’ approach lies in the development of moral capacity on the part of the individual; encouraging them, along Aristotelian lines, to engage in examination and reflection on their life and experience in order to come to a sense of what it is to live ethically and to inhabit an ‘ethical persona’. Thus moral sense and ethical expertise is developed from within. We believe that given the current organisational and institutional constraints of the public health curriculum, our short course will go some way to both provide future public health practitioners with a tool-box founded on our seven principles framework of public health ethics and also foster the development through its focus on experiential and problem-based learning, and the active application of case studies, of ‘inside out’ ethics.
